# Two cases of gastric mucosa-associated lymphoid tissue (MALT) lymphoma masquerading as follicular gastritis

**DOI:** 10.3332/ecancer.2019.933

**Published:** 2019-06-03

**Authors:** Masaya Iwamuro, Takehiro Tanaka, Kenji Nishida, Hiromitsu Kanzaki, Seiji Kawano, Yoshiro Kawahara, Tadashi Yoshino, Hiroyuki Okada

**Affiliations:** 1 Department of Gastroenterology and Hepatology, Okayama University Graduate School of Medicine, Dentistry and Pharmaceutical Sciences, Okayama 700-8558, Japan; 2 Department of Pathology, Okayama University Hospital, Okayama 700-8558, Japan; 3 Department of Pathology, Okayama University Graduate School of Medicine, Dentistry and Pharmaceutical Sciences, Okayama 700-8558, Japan; 4 Department of Endoscopy, Okayama University Hospital, Okayama 700-8558, Japan

**Keywords:** mucosa-associated lymphoid tissue lymphoma, gastrointestinal endoscope, gastric neoplasms, follicular gastritis, nodular gastritis

## Abstract

In this report, we describe two cases of extranodal marginal zone lymphoma of mucosa-associated lymphoid tissue (MALT lymphoma) of the stomach, which presented with multiple small, whitish nodules in the gastric body. The endoscopic appearance was similar to that of lymphoid follicular hyperplasia found in follicular gastritis or nodular gastritis. Both patients were positive for *Helicobacter pylori*, and the eradication treatment resulted in complete remission of the lymphoma. However, recurrence was noted in one patient. These cases indicate that, although infrequent, gastric MALT lymphoma can show a nodular appearance resembling that of follicular gastritis.

## Introduction

*Helicobacter pylori* (*H. pylori*) infection in the stomach leads to inflammation of gastric mucosa, which in turn leads to various types of alterations of the microscopic morphology. The process includes regenerative changes, intestinal metaplasia and foveolar hyperplasia [1]. In some patients, *H. pylori* infection causes the formation of prominent lymphoid follicles and mononuclear cell infiltration. This form of gastric inflammation has been endoscopically described as a nodular or diffuse miliary pattern of small elevations, identified typically in the antrum and occasionally extending to the whole gastric body [2–9]. This unique macroscopic feature was first reported as ‘gooseflesh skin-like’ gastric mucosa [2, 10] and is being increasingly called follicular gastritis or nodular gastritis in endoscopic practice. Although the term is not formally cited in the Sydney System for the classification of gastritis [2, 11], ‘nodularity’ is included as one of the features associated with active H. pylori infection in the Kyoto classification [2–14].

In this paper, we report two cases of extranodal marginal zone lymphoma of mucosa-associated lymphoid tissue (MALT lymphoma) of the stomach, which presented multiple small, whitish nodules resembling follicular gastritis. It was noteworthy that the nodularity was predominantly observed in the gastric body, rather than in the antrum. Biopsy examination revealed monomorphic proliferation of B-cells that were positive for CD20 and negative for CD3, CD10 and Cyclin D1. Lymphoepithelial lesions were also present, leading to the definitive diagnosis of gastric MALT lymphoma.

## Case report

### Case 1

A 41-year-old Japanese man underwent esophagogastroduodenoscopy screening. He had diabetes mellitus, hypertension and dyslipidaemia, and was receiving metformin, amlodipine and pravastatin. He had no history of gastrointestinal disease. The physical examination revealed no abnormalities and no evidence of peripheral lymphadenopathy. Laboratory findings demonstrated elevated levels of choline esterase (475 U/L), aspartate aminotransferase (54 U/L) and alanine aminotransferase (89 U/L), probably related to non-alcoholic fatty liver. The white blood cells count (9,090/μL) and eosinophil fraction (11.3%) were increased, but no atypical lymphocytes were identified in the peripheral blood. The patient’s serum was positive for anti-*H. pylori* immunoglobulin G antibodies, and his urea breath test was also positive.

**Figure 1.**
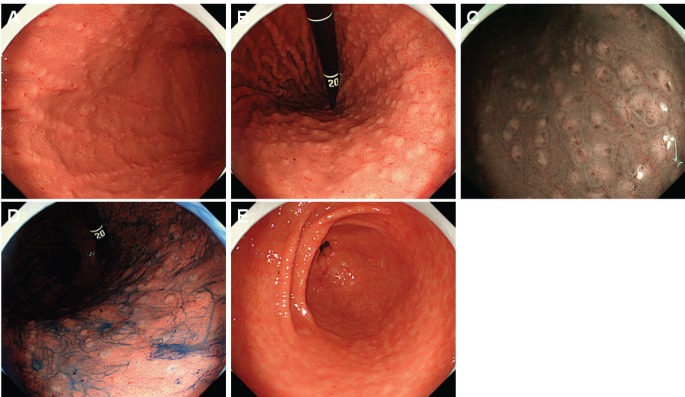
Esophagogastroduodenoscopy images of Case 1. (a) and (b): A diffuse miliary pattern of slightly whitish, small elevations was identified in the gastric body. (c): The elevations were emphasised on narrow-band imaging. (d): After indigo carmine spraying. (e): Granular appearance was not evident in the antrum.

**Figure 2.**
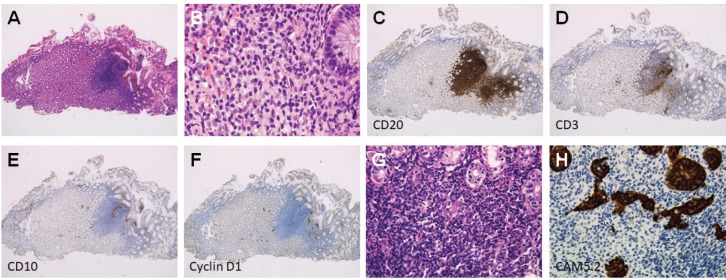
Pathological images of Case 1. (a): Follicle formation in a biopsy specimen. (b): Monomorphic lymphocytes. (c): Cells positive for CD20. (d): Negative for CD3. (e): CD10. (f): Cyclin D1. (g) and (h): Lymphoepithelial lesions.

Esophagogastroduodenoscopy showed a diffuse miliary pattern with slightly whitish, small elevations in the gastric body (Figure 1a and b). The multiple granular elevations were emphasised on narrow-band imaging (Figure 1c) and after indigo carmine spraying (Figure 1d). A mild atrophic change was noted in the gastric antrum compared with the gastric body, but the granular appearance was not evident (Figure 1e). A biopsy specimen from part of the small elevations of the gastric body revealed follicle formation (Figure 2a). Infiltrating lymphocytes within the follicle were monomorphic (Figure 2b) and positive for CD20 (Figure 2c), but they were negative for CD3 (Figure 2d), CD10 (Figure 2e) and Cyclin D1 (Figure 2f). Staining with haematoxylin and eosin (Figure 2g) and anti-human cytokeratin clone CAM5.2 showed lymphoepithelial lesions (Figure 2h). Fluorescence *in situ* hybridisation (FISH) analysis for t(11;18)(q21;q21) translocation revealed no fusion genes of baculoviral IAP repeat-containing protein 3 (BIRC3)-MALT1. On ^18^F-fluorodeoxyglucose positron emission tomography, no tracer uptake was noted. Colonoscopy and bone marrow biopsy revealed no lymphoma lesions as well. Consequently, the gastric lesion was diagnosed as stage I MALT lymphoma of the stomach.

**Figure 3.**
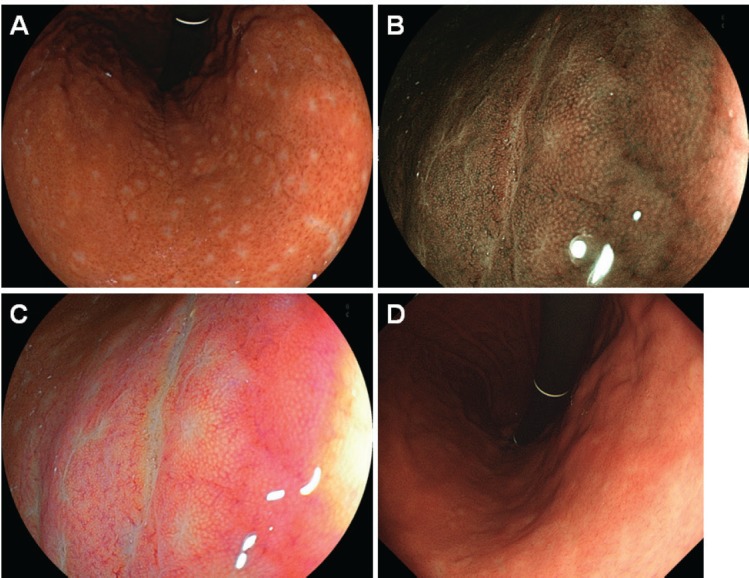
Esophagogastroduodenoscopy images of Case 1. (a)–(c): 3 months. (d): 45 months after eradication of *H. pylori*. Small elevations disappeared and whitish spots could be partly observed in the gastric body. (b): Magnifying observation with narrow-band imaging and (c) linked colour imaging also showed that the elevations regressed. (d): Esophagogastroduodenoscopy performed 45 months after eradication of *H. pylori* showed vague whitish spots in the gastric body.

Because the patient tested positive for *H. pylori*, eradication treatment was attempted. Esophagogastroduodenoscopy performed 3 months after the completion of eradication treatment showed disappearance of the small elevations, while whitish spots were partly observed in the gastric body (Figure 3a–c). Elimination of neoplastic cells was pathologically confirmed on the biopsy specimens. No recurrence was documented for 45 months since complete remission was achieved. Esophagogastroduodenoscopy performed 45 months after achieving complete remission showed that the whitish spots had almost disappeared (Figure 3d).

### Case 2

A 54-year-old Japanese woman underwent esophagogastroduodenoscopy for screening purposes. She had been receiving amlodipine for the treatment of hypertension. The physical examination revealed no abnormalities. The laboratory findings demonstrated elevated levels of glutamyl transpeptidase (111 U/L). Other blood chemistry and complete blood count were within the normal ranges. The test for serum anti-*H. pylori* immunoglobulin G antibodies showed positive results.

**Figure 4.**
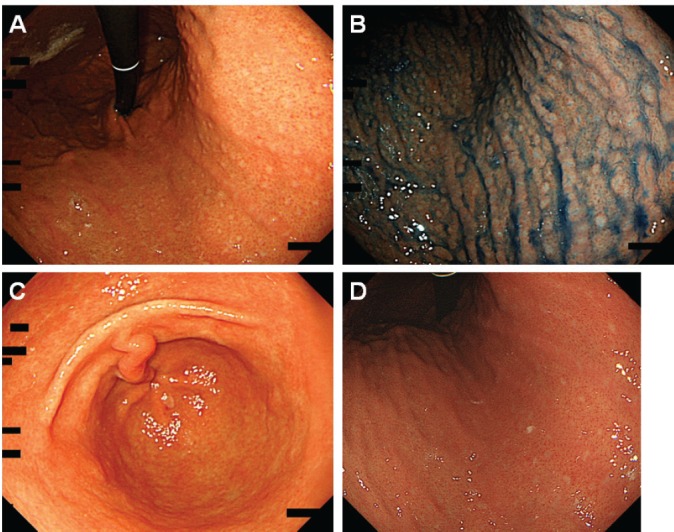
Esophagogastroduodenoscopy images of Case 2. [(a) and (b): After indigo carmine spraying] Slightly whitish, small, multiple elevations can be seen in the lesser curvature of the gastric body. (c) and (d): The small elevations regressed 5 months after *H. pylori* eradication.

**Figure 5.**
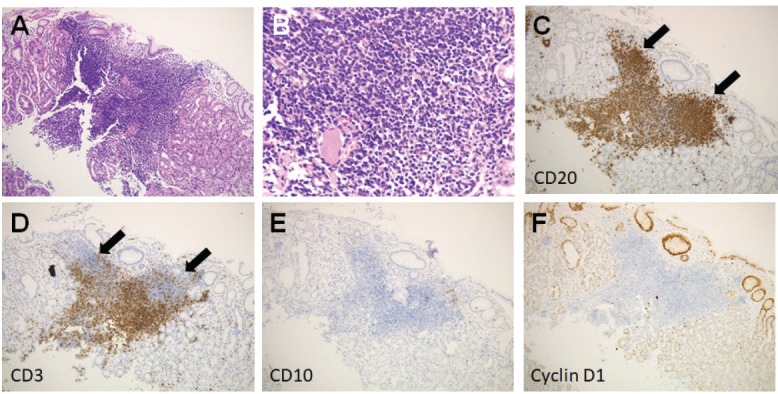
Pathological images in Case 2. (a) and (b): Haematoxylin and eosin staining of a biopsy specimen. (c): Cells positive for CD20. (d): Negative for CD3. (e): CD10. (f): Cyclin D1.

**Figure 6.**
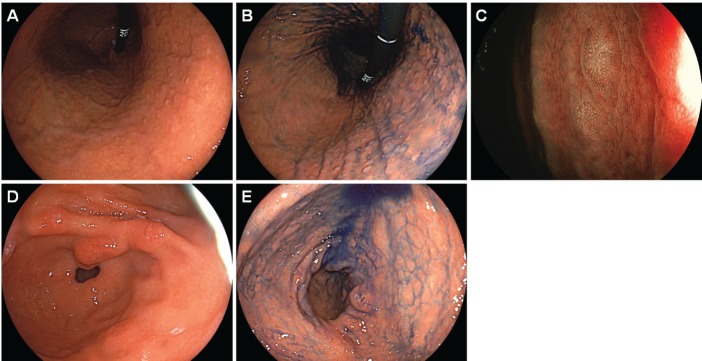
Esophagogastroduodenoscopy images of Case 2 performed 29 months after the initial examination. [(a) and (b): After indigo carmine spraying] Miliary appearance re-emerged in the gastric body. (c): Magnifying observation with narrow-band imaging showed small elevations. [(d) and (e): After indigo carmine spraying] Granular appearance was not evident in the antrum.

**Figure 7.**
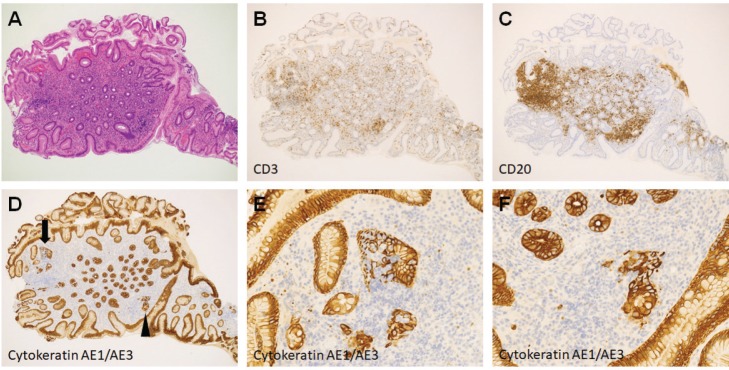
Pathological images of Case 2. (a) Haematoxylin and eosin staining of a biopsy specimen. (b): Cells negative for CD3. (c) Positive for CD20. [(d) arrow and arrowhead]: Lymphoepithelial lesions were noted in cytokeratin AE1/AE3 staining. (e): Magnified views of arrow. (f): Arrowhead.

Esophagogastroduodenoscopy showed multiple slightly whitish, small elevations in the lesser curvature of the gastric body (Figure 4a and b). The granular appearance was not evident in the gastric antrum (Figure 4c). Biopsy from the elevation revealed diffuse infiltration of monomorphic lymphocytes, which were predominantly positive for CD20 on immunochemical analysis (Figure 5). FISH analysis for t(11;18)(q21;q21) translocation revealed no fusion genes of BIRC3-MALT1. Gastric MALT lymphoma was highly suspected, but definitive diagnosis could not be established, because no prominent lymphoepithelial lesion was identified. The patient underwent eradication treatment for *H. pylori*. Esophagogastroduodenoscopy performed 5 months after *H. pylori* eradication revealed regression of small elevations (Figure 4c). Monomorphic lymphocytes and *H. pylori* disappeared from the biopsy specimens. However, esophagogastroduodenoscopy performed 24 months after the initial examination showed re-emergence of miliary appearance in the gastric body. Infiltration of monomorphic B-cells was noted in the biopsy specimens, and the lesion was pathologically diagnosed as probable MALT lymphoma of the stomach. Five months later, multiple granular elevations remained on the lesser curvature of the gastric body (Figure 6). Pathological analysis revealed infiltration of small- to medium-sized monomorphic B-cells (Figure 7a–c) showing prominent lymphoepithelial lesions (Figure 7d–f). Thus, the definitive diagnosis of gastric MALT lymphoma was established. The patient underwent computed tomography, colonoscopy and bone marrow biopsy, and no lymphoma lesions were noted. Radiotherapy was planned for the treatment of gastric MALT lymphoma.

## Discussion

In this report, we describe two cases of gastric MALT lymphoma presenting with a diffuse miliary appearance with slightly whitish and small elevations. The endoscopic features were similar to those of *H. pylori*-associated gastritis, known as follicular gastritis or nodular gastritis. It was noteworthy that the nodularity was predominant in the gastric body in the present two cases, whereas it is generally observed in the gastric antrum in follicular gastritis. As shown in Figures 2 and 5, the nodularity probably resulted from neoplastic follicle formation by MALT lymphoma cells.

Although gastric MALT lymphomas present a variety of macroscopic features, MALT lymphoma resembling follicular gastritis is infrequent. According to the previously reported classification of this disease [15], we retrospectively reviewed the data of 82 patients with gastric MALT lymphoma diagnosed at our institution. The endoscopic features of these patients included whitish lesions (*N* = 38, 46.3%), reddish or erosive lesions (*N* = 15, 18.3%), ulcers (*N* = 8, 9.8%), elevated lesions (*N* = 6, 7.3%), cobblestone appearance (*N* = 6, 7.3%), mucosal swelling or oedematous lesions (*N* = 4, 4.9%), depressed lesions resembling early gastric cancer (*N* = 3, 3.7%) and other lesions (*N* = 2, 2.4%). The last group comprised the two cases presented in this case report. Thus, at our institution, MALT lymphoma resembling follicular gastritis represented 2.4% of all gastric MALT lymphomas. Zullo *et al* [16, 17] proposed an updated endoscopic classification of gastric MALT lymphoma, as follows: ulcerative (single/multiple ulcerations or erosions), exophytic (irregular or polypoid mass), hypertrophic (large gastric fold or nodular mucosa), petechial haemorrhage (multiple mucosal petechial haemorrhages), normal (absence of macroscopic lesions) and mixed (a combination of more patterns). When whitish MALT lymphoma lesions without haemorrhages were subclassified as ‘normal,’ the endoscopic features of the 82 patients were normal (*N* = 32, 39.0%), ulcerative (*N* = 19, 23.2%), petechial haemorrhage (*N* = 13, 15.9%), hypertrophic (*N* = 11, 13.4%) and exophytic (*N* = 7, 8.4%). According to this classification, the two cases presented in this case report are classified as ‘hypertrophic.’

To our knowledge, only two cases of gastric MALT lymphoma showing similar endoscopic features to those of follicular gastritis have been reported (Table 1). Cheng *et al* [18] described a 49-year-old woman with gastric MALT lymphoma, presenting as fine granular mucosal change in the lower gastric body and antrum. The authors noted that the eradication of *H. pylori* resulted in complete remission. Lee *et al* [19] reported another case of a 12-year-old girl with gastric MALT lymphoma masquerading as follicular gastritis on endoscopy. Pathological features of MALT lymphoma improved after the eradication of *H. pylori* in this patient as well. Previously reported cases and the results of our review indicate that, although infrequent, gastric MALT lymphoma emerges with similar appearance to that of follicular gastritis. Owing to the rarity of this type of MALT lymphoma, the biological characteristics of gastric MALT lymphoma masquerading as follicular gastritis have not been elucidated. However, as the present two patients and previously reported patients were positive for *H. pylori* infection, we speculate the possible involvement of *H. pylori* in lymphoid follicle formation in the gastric body, which leads to a nodular or miliary pattern of small elevations.

**Figure 8.**
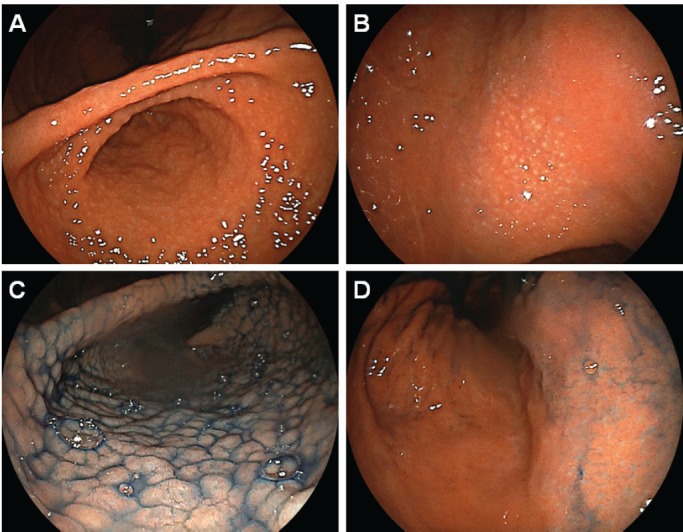
Typical endoscopic images of follicular gastritis. In a 21-year-old Japanese woman with H. pylori infection, (a) miliary appearance was noted in the gastric antrum (b) in the body. (c): After indigo carmine spraying, nodularity was predominantly observed in the antrum. (d): Rather than in the body.

Intact gastric mucosa is believed to inherently lack lymphocytic aggregates. *Helicobacter pylori* infection in the stomach induces chronic inflammation, leading to the development of lymphoid follicles [5, 20]. Emerged lymphoid follicles are called MALT. As described above, in some patients, lymphocytic aggregates and follicles in the gastric mucosa cause a granular appearance predominantly in the antrum, which is termed as follicular gastritis or nodular gastritis (Figure 8). The reported prevalence of follicular gastritis was 0.7%–2.9% [4, 6, 9, 21]. In contrast to the predominant involvement of the antrum in follicular gastritis, the present two cases with MALT lymphoma had prominent nodularity in the gastric body and nodularity was less obvious in the antrum. Therefore, we consider that endoscopists should take biopsy samples to differentiate the two entities when they observe a nodular or miliary pattern of small elevations in the gastric body.

The difficulty in differentiating gastric MALT lymphoma from follicular gastritis based on pathological features has been previously reported [18, 22–24]. Hummel *et al* [24] investigated B-cell clonality in cases with chronic gastritis (Wotherspoon scores 1 and 2, *N* = 53), overt gastric MALT lymphoma (Wotherspoon score 5, *N* = 26) and ambiguous histology (Wotherspoon scores 3 and 4, *N* = 18). The authors noted that B-cell clonality was found in 1/53 cases of chronic gastritis (1.9%), 24/26 cases of overt lymphoma (92.3%) and 4/18 cases with ambiguous histology (22.2%). Similarities and ambiguities in pathologic and molecular biologic features between the two disease entities indicate a possible transition from chronic gastritis to lymphoma during the pathogenesis of gastric MALT lymphoma [6, 9, 24–27]. The present two cases show morphological similarities of MALT lymphoma with follicular gastritis on endoscopy, which may be another evidence for this issue.

*Helicobacter pylori* eradication is the appropriate first-line treatment strategy for follicular gastritis. Morphological regression of nodular appearance on endoscopy and decrease in number and size of the lymphoid follicles on pathology after a successful *H. pylori* eradication has been previously reported [2, 6, 9, 20, 21]. Even monoclonal B-cell populations, if they are detected in follicular gastritis, disappear after *H. pylori* eradication [9]. In the present two cases, nodularity of the gastric mucosa disappeared after *H. pylori* eradication and the gastric lesions turned to be whitish spots in both cases. However, although complete remission was obtained for 34 months in Case 1, re-emergence of miliary appearance was identified in Case 2 even after *H. pylori* eradication.

Different outcomes have been reported for several subsets of gastric MALT lymphoma. For example, it is well known that patients with t(11;18) translocation are resistant to *H. pylori* eradication [28, 29]. In our previous work, we have revealed that progression or relapse tended to be more frequent in patients with extra copies of *MALT1* [30]. Conversely, MALT lymphoma with increased plasma cell differentiation shows favourable treatment response [31, 32]. Based on the outcome observed in Case 2 of the present report, careful follow-up may be necessary in patients with gastric MALT lymphoma resembling follicular gastritis to detect relapse. Further investigation is required to reveal treatment response and long-term outcomes of these patients.

## Conclusion

We presented two cases of gastric MALT lymphoma showing a nodular appearance. Although presentation with such morphology is likely to be infrequent, the present two cases indicate that gastric MALT lymphoma may present a nodular appearance resembling that of follicular gastritis. Biopsy examination should be performed when a nodular or miliary pattern of small elevations is observed, particularly in the gastric body rather than in the antrum.

## Conflict of interest

The authors declare that they have no conflict of interests regarding the publication of the paper.

## Funding declaration

The authors received no specific funding for this work.

## Figures and Tables

**Table 1.** Reported cases with gastric MALT lymphoma resembling follicular gastritis.

No.	Author	Age	Sex	Macroscopic features	Involved area	Treatment	Outcome
1	Cheng *et al* [18]	49	F	Fine granular mucosal change	Lower gastric body and antrum	Eradication of *H. pylori*	Complete remission
2	Lee *et al* [19]	12	F	Diffuse carpet-like mucosal nodularity	Gastric antrum	Eradication of *H. pylori*	Complete remission
3	Present case	41	M	Miliary pattern with slightly whitish, small elevations	Gastric body	Eradication of *H. pylori*	Complete remission
4	Present case	54	F	Slightly whitish, small, multiple elevations	Gastric body	Eradication of *H. pylori*, radiotherapy	Temporal regression and relapse

F, female; M, male.
